# Exome Sequencing Identifies Variants in *MLH1* and *ERBB2* as Potential Cancer‐Predisposing Factors in Familial Early‐Onset Colorectal Cancer

**DOI:** 10.1002/kjm2.70166

**Published:** 2026-01-02

**Authors:** Behnaz Bagheri, Neda Mohsen‐Pour, Najmeh Salehi, Pardis Ketabi Moghadam, Adel Zeinalpour, Amir Sadeghi, Samira Kalayinia, Nayeralsadat Fatemi

**Affiliations:** ^1^ Basic and Molecular Epidemiology of Gastrointestinal Disorders Research Center, Research Institute for Gastroenterology and Liver Diseases Shahid Beheshti University of Medical Sciences Tehran Iran; ^2^ Zanjan Pharmaceutical Biotechnology Research Center Zanjan University of Medical Sciences Zanjan Iran; ^3^ School of Biology, College of Science University of Tehran Tehran Iran; ^4^ Gastroenterology and Liver Diseases Research Center, Research Institute for Gastroenterology and Liver Diseases Shahid Beheshti University of Medical Sciences Tehran Iran; ^5^ Department of General Surgery, School of Medicine Shahid Beheshti University of Medical Sciences Tehran Iran; ^6^ Cardiogenetic Research Center Rajaie Cardiovascular Institute Tehran Iran

**Keywords:** colorectal cancer, early‐onset, *ERBB2*, *MLH1*, whole exome sequencing

## Abstract

Colorectal cancer (CRC) has raised considerable health concerns worldwide, with increasing incidence rates, specifically among younger populations. Despite remarkable progress in diagnosing and treating various diseases, the genetic basis of CRC remains only partially understood. This paper aims to examine novel genetic variants associated with CRC in Iranian patients. We performed whole‐exome sequencing (WES) in two Iranian families with a history of early‐onset CRC. Candidate variants were validated by Sanger sequencing and assessed for segregation. Pathogenicity was evaluated through comprehensive in silico analysis and the application of ACMG/AMP guidelines. Analysis revealed two clinically significant variants. In Family 1, we identified a heterozygous stop‐gain variant in *MLH1* (c.1043T>A, p.Leu348), a known pathogenic mutation consistent with Lynch syndrome. In Family 2, we discovered a previously undocumented heterozygous missense variant in *ERBB2* (c.2268G>T, p.Arg756Ser). Through a detailed ACMG assessment, this *ERBB2* variant was classified as Likely Pathogenic based on its location in a critical tyrosine kinase domain, absence from population databases, and concordant deleterious in silico predictions. WES offers a deeper understanding of CRC genetics, suggesting potential biomarkers with promising applications for early diagnosis and targeted treatments, eventually improving patient‐related outcomes. The results of this study underscore the significant contribution of genetic screening to the well‐being of high‐risk families and offer valuable insights for targeted therapeutic approaches.

**Abbreviations**
BQSR: base quality score recalibration
CADD: combined annotation dependent depletion
COSMIC: catalog of somatic mutations in cancer
CRC: colorectal cancer
gnomAD: genome aggregation database
HGVS: human genome variation society
Mlh1: MutL Homolog 1
MMR: mismatch repair
MSI: microsatellite instability
mTOR: mammalian target of rapamycin
NGS: next‐generation sequencing
PCR: polymerase chain reaction
PI3K: Phosphoinositide 3 kinase
PKC: protein kinase C
PLC: phospholipase C
RCGLD: research center for gastroenterology and liver diseases
WES: whole‐exome sequencing

## 1 Introduction

Colorectal cancer (CRC) is currently ranked as the third most commonly reported malignant disease and the second leading cause of cancer‐related deaths worldwide [1]. It is projected that CRC cases will increase by 60% by 2030, resulting in more than 2.2 million new diagnoses and 1.1 million fatalities [2]. While extensive research has been conducted on CRC‐associated variants in Western countries, there remains a significant gap in knowledge regarding Middle Eastern cohorts, particularly those affected by early‐onset CRC [3]. Recent epidemiological research has reported a significant increase in the prevalence of CRC, particularly among younger populations under the age of 55, and has revealed that cases are being diagnosed at more advanced stages [4].

CRC represents a highly heterogeneous type of cancer, with inherited genetic factors playing a critical role in its occurrence and progression. Similar to many other cancers, CRC is influenced by both environmental factors and genetic predispositions [5]. Although significant advancements in diagnostic, prognostic, and therapeutic strategies have led to a notable reduction in CRC‐related mortality, these declines have primarily been observed in developed countries [6]. The molecular mechanisms underlying CRC are complex and not yet fully understood. Therefore, the causal genes responsible for a substantial portion of hereditary colorectal adenomas and early‐onset CRC remain to be fully elucidated [7]. Despite the significantly higher incidence of early‐onset CRC in the Middle East compared to Western regions, rare CRC‐associated genetic variants have largely remained underexplored in these populations.

This gap underscores the need for closer investigations into the susceptibility‐related rare genetic variants of CRC in the Middle East. By examining cancer predisposition genes through pathogenic rare variants, we can substantially enhance our understanding of the genetic foundations of CRC in these populations. This approach may reveal valuable.

preventive markers and advance precision or personalized medicine [8]. Although definitive causes of CRC have yet to be identified, recent advancements in next‐generation sequencing (NGS), specifically whole exome sequencing (WES), have significantly transformed translational biomedicine [9]. These technologies enable researchers to analyze multiple genes simultaneously while substantially reducing costs.

Here, we utilized WES of two unrelated families diagnosed with CRC at a young age. We searched for novel or rare pathogenic variants that could elucidate the early onset of CRC. Additionally, to assess the pathogenic potential of one of the candidate variants, we conducted in silico protein structure modeling.

## 2 Materials and Methods

### 2.1 Ethics Approval and Consent to Participate

This study was conducted following the Declaration of Helsinki and received approval from the Ethics Committee of the Research Center for Gastroenterology and Liver Diseases (RCGLD) (IR.SBMU.RIGLD.REC.1404.013). All participants provided written informed consent for their involvement in the research and for the publication of the findings.

### 2.2 Study Patients and Clinical Evaluation

**FIGURE 1:**
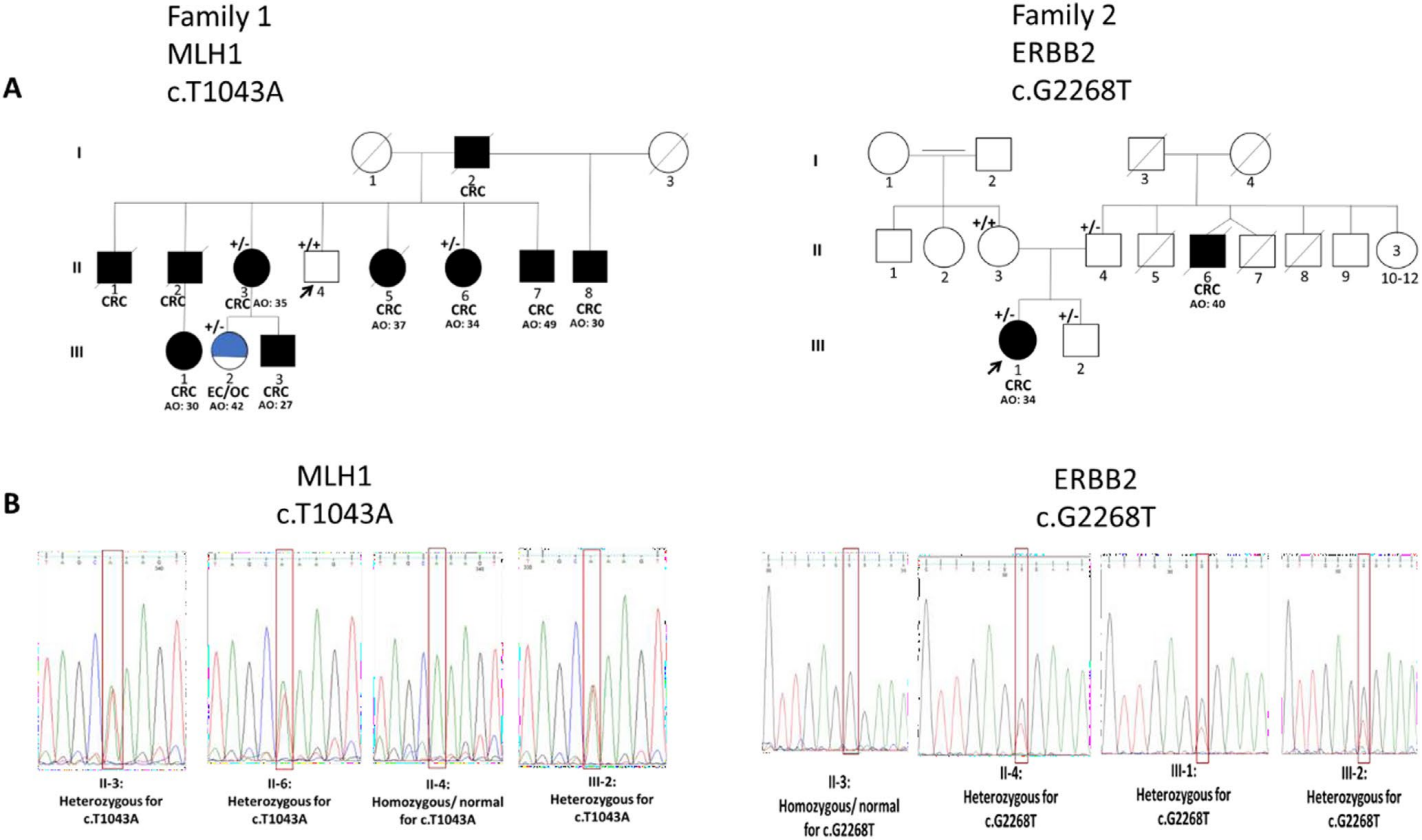
*MLH1* c.1043T>A (p.L348X, NM_000249), and *ERBB2* c.2268G>T (p. R756S, NM_001289937) variants co‐segregating in family 1 and family 2. (A) Family 1 and family 2 pedigrees and co‐segregation of the two identified variants. (B) Sequencing chromatograms showing the variant and wild‐type alleles for the two identified variants. Filled squares indicate those affected by CRC. The arrow indicates the proband. +/−, variant carrier; +/+, wild type; AO, age of onset; CRC, colorectal cancer; EC, endometrium cancer; OC, ovarian cancer.

The study focused on two Iranian families with a history of CRC. Figure 1A illustrates the pedigrees of these families, along with the patients' characteristics. Blood samples were collected from available family members, both affected and unaffected, and their medical records were thoroughly reviewed. All selected patients had been diagnosed with CRC at or before the age of 49 (range: 27–49).

### 2.3 Whole‐Exome Sequencing Data Analysis

WES was conducted on genomic DNA extracted from peripheral blood samples of individuals II‐6 (the proband's sister) in Family 1 and III‐1 in Family 2 to identify the causative gene variants.

The fragmented DNA was captured using the MGIEasy Exome Capture V5 Probe Set. Subsequently, paired‐end sequencing of the exon‐enriched libraries was carried out on the MGI DNBSEQ‐G400 platform, achieving a mean target coverage of 100×. The sequencing reads were aligned to the human genome reference (hg19/NCBI) using Minimap2. Variant identification followed GATK Best Practices, which included marking duplicates, performing base quality score recalibration (BQSR), and calling variants using HaplotypeCaller with known variant sites from the dbSNP (v151) and the 1000 Genomes Project.

### 2.4 Variant Validation and Co‐Segregation Analysis

Candidate variant segregation from exome data was evaluated by polymerase chain reaction (PCR)‐based Sanger sequencing. The primers were designed and validated using the Primer3 software (v0.4.0; 29) and BLAST (https://www.ncbi.nlm.nih.gov/tools/primer‐blast/index.cgi?LINK_LOC=BlastHome). The primer sequences used in this study are listed in Table 1. Conventional PCR was performed using Taq DNA Polymerase Master Mix (Ampliqon, Odense, Denmark). PCR was performed at 94°C for 4 min, followed by 32 cycles at 94°C for 30 s, with specific annealing temperatures for *ERBB2* and *MLH1* for 30 s, and an extension at 72°C for 30 s.

**TABLE 1:** Primer sequences.

Primer	Sequence (5′–3′)
*MLH1*‐fwd	TATCCCGTCTTAGTCCCATGG
*MLH1*‐rev	CTGGGGTTGCTGGAAGTAGGT
*ERBB2*‐fwd	TGGCACAGTCTACAAGGTCAGG
*ERBB2*‐rev	CAGCCTCCCAACCATCACA

A final extension step was carried out for 5 min at 72°C. The PCR products were assayed using the 3130xl genetic analyzer (Applied Biosystems). We employed Sequencing Analysis v5.2 (Applied Biosystems) and FinchTV v1.5.0 (Geospiza Inc.) to determine the sequences of the PCR products and validate the candidate variants.

### 2.5 3D Structure Prediction

Because no fully determined three‐dimensional (3D) structures of MLH1 and ERBB2 have been experimentally determined, we used the AlphaFold3 web server (https://alphafoldserver.com/) to predict their structures. The FASTA sequences of the canonical isoforms were extracted from the UniProt database, utilizing the accession IDs P40692 and P04626 for MLH1 and ERBB2, respectively. The native and mutant sequences were uploaded to the AlphaFold3 web server. To investigate interactions between the native and mutant forms of MLH1 and PMS2, the 3D structures of these complexes were modeled using AlphaFold3. The most confident structures generated for each model variant were chosen for further visualization and analysis. The binding affinities of the interactions were determined using PRODIGY. All structures were visualized using VMD software.

## 3 Results Clinical Data

We enrolled two Iranian families (Family 1 and Family 2) with CRC. In Family 1, the proband (II‐4) is a 68‐year‐old male who undergoes annual colonoscopy screenings. The proband's father (I‐2) died at the age of 52 and, according to the proband, had experienced acute bowel problems, possibly indicating undiagnosed CRC. The proband's mother (I‐1) passed away at 62 years of age due to a heart attack. The age of CRC onset in the proband's sister (II‐5) was 37 years. She had liver metastases and has since passed away. The proband's brothers (II‐1 and II‐2) also succumbed to CRC.

The affected individuals spanned three generations. In total, ten individuals in Family 1 were diagnosed with CRC at high risk for Lynch syndrome, comprising four females (II‐3, II‐5, II‐6, and III‐1) and six males (I‐2, II‐1, II‐2, II‐7, II‐8, and III‐3).

In Family 2, the proband (III‐1) is a 34‐year‐old female who was diagnosed with CRC at the age of 34. The proband's brother (III‐2) had one benign polyp, while her father (II‐4) had multiple benign polyps. The proband's uncle (II‐6) passed away from CRC at the age of 70. Additionally, there is a reported history of CRC in the proband's maternal lineage, including cases among maternal uncles and cousins. Table 2 presents the clinicopathologic characteristics of the CRC cases evaluated in this study.

**TABLE 2:** The clinic‐pathological characteristics of CRC patients.

Patient ID	Age at diagnosis (year)	Sex	Tumor location	Cancer stage at diagnosis (TNM)	Pathological tumor type and grade	Treatment history	Treatment response outcomes	MMR IHC interpretation	PCR‐based MSI testing
Family 1‐(II‐6)	34	Female	Rectum	IIIB (T3, N1, M0)	Poorly differentiated adenocarcinoma	Surgery (left colectomy) + adjuvant	No evidence of disease	MMR deficient (MLH1/PMS2 loss)	MSI‐H
Family 2‐(III‐1)	34	Female	Distal colon	IIA (T3, N0, M0)	Moderately differentiated adenocarcinoma	Chemotherapy surgery (resection)	No evidence of disease	MMR proficient	MSS

Abbreviations: IHC, immunohistochemistry; MMR, deficiency by IHC is defined by the loss of expression of one or more MMR proteins (MLH1, MSH2, MSH6, PMS2); MMR, mismatch repair; MSI, microsatellite instability; MSI‐H, microsatellite instability‐high; MSS, microsatellite stable; PCR, polymerase chain reaction; TNM, tumor, node, metastasis staging system.

## 4 Pathogenic Variant Identification and Validation

Genetic Sequence analysis revealed a pathogenic variant in Family 1: c.1043T>A (p.L348X, NM_000249), located in exon 12 of the *MLH1* gene. This variant results in the substitution of leucine at position 348 with a premature stop codon (Table 3). The proband (II‐4) was wild‐type (non‐carrier) for MLH1 (c. 1043T>A, p.L348X). Sanger sequencing confirmed the presence of this variant in a heterozygous state in individuals II‐3, II‐6, and III‐2, as shown in Figure 1B.

**TABLE 3:** Overview of the variants identified by WES and In silico prediction analysis.

Genes	Variant functional pathway	Position	COSMIC ID	HGVS	Variant type	Protein change	gnomAD	CADD	SIFT	PolyPhen‐2	REVEL	ClinVar classification
*MLH1*	DNA mismatch repair protein, C‐terminal domain	chr3: 37067132	No data	c.1043T>A‐NM_0002 49	Stop gain loss‐of‐function	Leucine3 48×	No data	37	Tolerated (1)	—	0.193 (uncertain)	Pathogenic
*ERBB2*	Tyrosine‐kinase type	chr17: 37880224	COSM1025 801	c.2268G>T‐NM_0012 89,937	Missense likely gain‐of‐function mutation	Arginine 756 Serine	No data	26.4	Deleterious (0.006)	Probably damaging (1)	0.599 (pathogenic)	VUS

Abbreviations: CADD, combined annotation dependent depletion; COSMIC, catalog of somatic mutations in cancer; gnomAD, the Genome Aggregation Database; HGVS, the human genome variation society; PolyPhen‐2, polymorphism phenotyping version 2; REVEL, rare exome variant ensemble learner; SIFT, sorting intolerant from tolerant; VUS, variants of uncertain significance.

Family 2 was found to carry a previously undocumented heterozygous missense variant in the *ERBB2* gene, c.2268G>T (p. R756S, NM_001289937), located within the critical tyrosine‐kinase domain of exon 19. In silico tools predict a deleterious effect of this missense change (Table 3). The presence of this c.2268G>T variant in the affected proband (III‐1) was confirmed through Sanger sequencing. Segregation analysis within the family revealed that the proband's unaffected father (II‐4, age 68) and unaffected brother (III‐2, age 42) were also heterozygous carriers. At the same time, the mother (II‐3) was homozygous for the wild‐type allele (Figure 1B). The presence of unaffected carriers indicates incomplete penetrance, a recognized phenomenon in hereditary cancer syndromes. Despite this limited segregation evidence, the variant was classified as Likely Pathogenic based on the cumulative weight of the following ACMG/AMP criteria: its absence in population databases (PM2), location within a critical and well‐established tyrosine kinase domain mutational hotspot (PM1), and concordant deleterious predictions from multiple in silico algorithms (PP3). Furthermore, its documentation as a recurrent somatic mutation in the COSMIC database (COSM1025801) provides independent, supporting evidence of its oncogenic potential. The substitution of a positively charged, bulky arginine with a polar, uncharged serine at this conserved position is predicted to disrupt the kinase's ATP‐binding site and is consistent with a gain‐of‐function mechanism, as observed in other oncogenic ERBB2 mutations. These observations suggest that the variants c. 1043T>A (p.L348X, NM_000249) in *MLH1* and c.2268G>T (p.R756S, NM_001289937) in the *ERBB2* gene may be responsible for CRC in these families.

## 5 The Structural Impact of MLH1 and ERBB2 Variants

**FIGURE 2:**
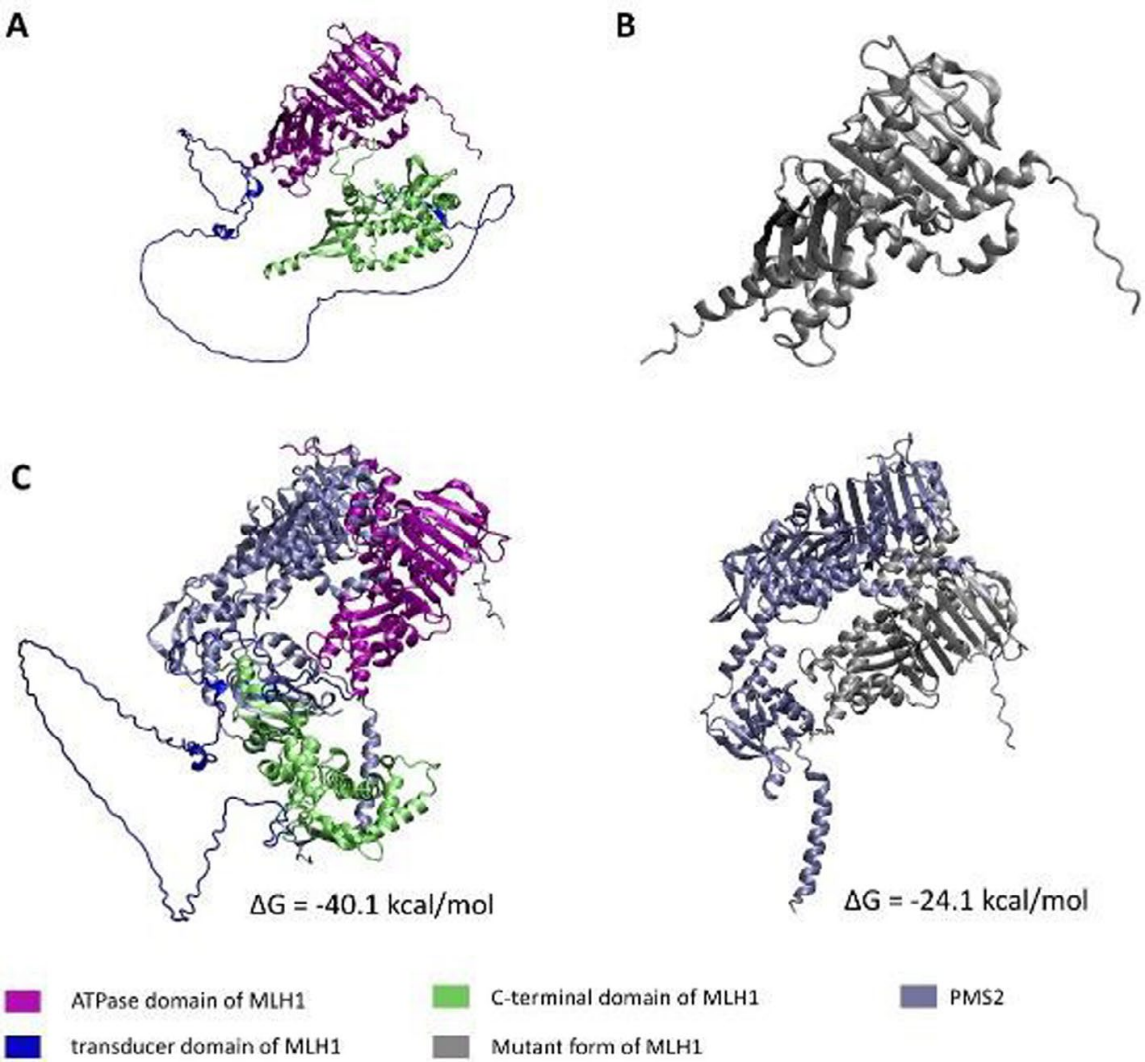
MLH1 3D structural model. (A) The 3D structural model of MLH1 in its native form, and (B) in the L348X mutant form. (C) The complex structure of MLH1–PMS2 in both the native and mutant forms of MLH1. Only the high‐confidence regions of the PMS2 protein structure are shown.

The complete structure of MLH1 was predicted using AlphaFold3, revealing its domains: an ATPase domain involved in ATP binding and hydrolysis, a transducer domain, and the C‐terminal region (Figure 2A). The L348X variant introduces a premature stop codon, replacing leucine at position 348 with a termination signal, resulting in a truncated MLH1 protein (Figure 2B). The C‐terminal domain, which is lost due to this variant, contributes to the endonuclease active site and is critical for interaction with PMS2, a key partner in the formation of the MutLα complex essential for DNA mismatch repair [10]. The free energy of the MLH1–PMS2 interaction increased from −40.1 kcal/mol in the MLH1 native form to −24.1 kcal/mol in the MLH1 mutant form, indicating reduced complex stability (Figure 2C).

Variants in the C‐terminal domain have been shown to disrupt MLH1 function and contribute to tumorigenesis by unveiling mechanisms of pathogenicity and clarifying their role in cancer progression [11, 12]. Loss of MLH1 function results in microsatellite instability (MSI), a hallmark of deficient DNA mismatch repair. MSI leads to the accumulation of variants, particularly in genes involved in cell cycle regulation, apoptosis, and growth signaling pathways, thereby promoting tumorigenesis [13].

**FIGURE 3:**
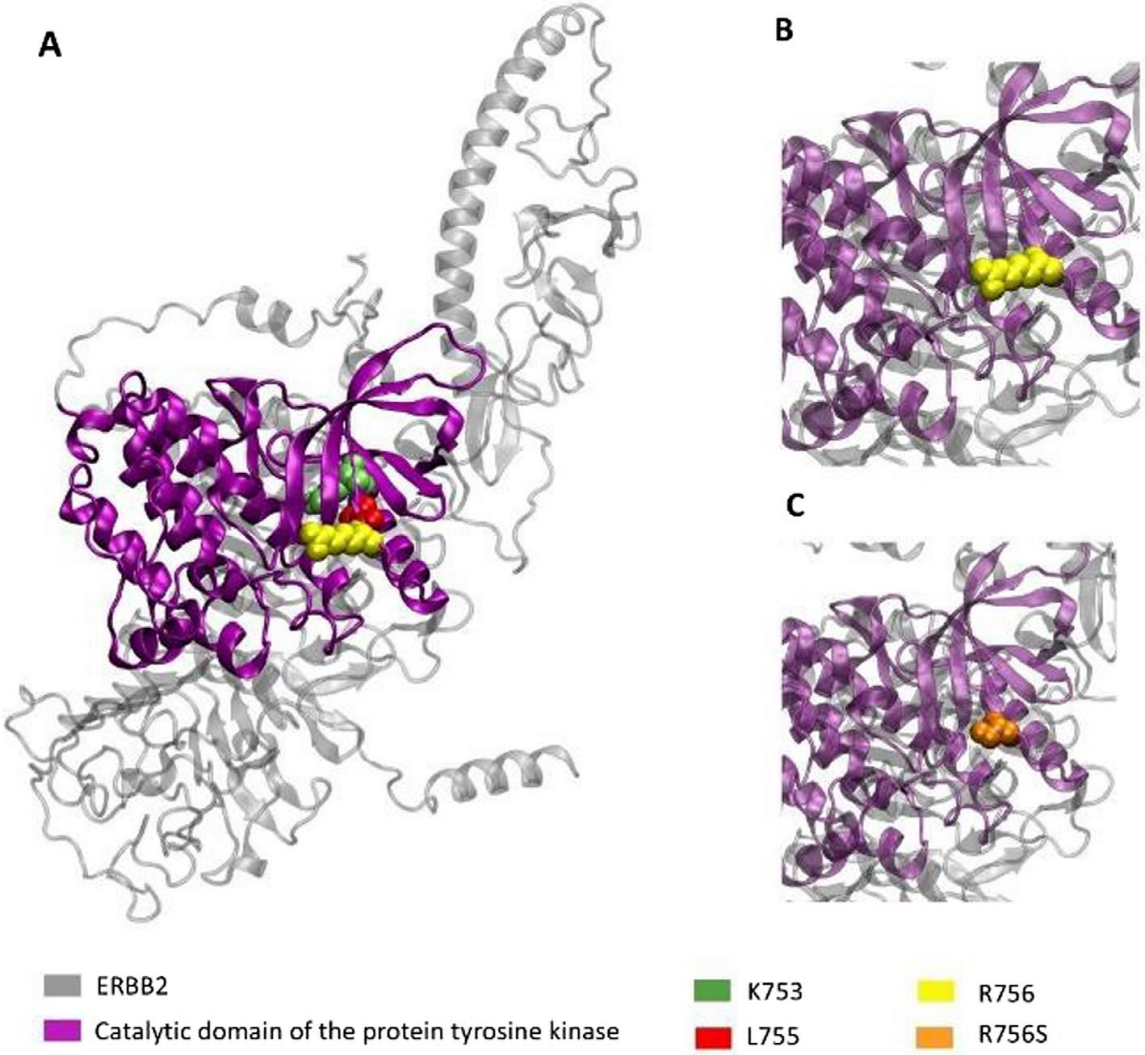
ERBB2 3D structural model. (A) The 3D structural model of ERBB2 in its native form, with high‐confidence residues shown. Close‐up view of the (B) native and (C) mutant residues at position 756 in the ERBB2 structure.

The 3D structure of ERBB2 was predicted using AlphaFold. The R756S variant is located within the catalytic domain of the protein tyrosine kinase (Figure 3), which plays a critical role in ERBB2 signaling and activity. In this variant, an arginine, a large positively charged amino acid, is replaced by a serine, a small uncharged amino acid. The mutated residue R756 is positioned near key functional sites. K753 is part of the ATP‐binding site, and this variant has been found in breast cancer [14]. Additionally, R756 is located near L755, a residue where the L755S variant has been frequently reported in various cancers [15]. L755S is associated with resistance to tyrosine kinase inhibitors, such as lapatinib, which is commonly used to treat ERBB2‐positive breast cancer [16]. Although R756S is less frequently reported than L755S, its location suggests that it may similarly affect kinase function or drug binding.

## 6 Discussion

CRC is the second most prevalent cancer in men and third in women, a heterogeneous disease involving multistep stages that represents 10% of all cancers worldwide. CRCs present a remarkably complex biologic profile, complicated by the lack of established known causes [17].

Molecular profiling offers a non‐invasive, rapid diagnostic approach using high‐throughput technologies such as NGS, which identifies hotspot gene variants that serve as high‐risk driver genes for detecting CRC cases. Genetic predisposition has been shown to make significant contributions to the development of CRC. Although extensive data are available on *RAS*/*RAF*/*PI3KA* and *TP53* gene variants, our understanding of less commonly mutated genes primarily depends on genomic‐scale analyses conducted on a relatively small group of CRC samples.

NGS has facilitated the discovery of several novel genes contributing to the CRC predisposition, among which *RPS20*, *POLE*, *POLD1*, *AXIN2*, *NTHL1*, *MSH3*, *RNF43*, *GREM1*, *MLH1*, and *ERBB2* can be mentioned as some examples [5, 18, 19]. One of the biggest challenges is estimating the penetrance of these genetic variants, which necessitates further research to validate existing relationships.

This paper may add to the expanding literature linking genetic variants to CRC, specifically focusing on the characterization and contributions of *MLH1* and *ERBB2*. As highlighted by relevant studies, the discovery of *MLH1* variants, particularly in the context of mismatch repair (MMR) deficiency, confirms that hereditary CRCs and Lynch syndrome (OMIM #120435) are associated. In this study, Family 1, carrying the *MLH1* variant (c.1043T>A, p.L348X), distinctly demonstrates that this variant segregates with early‐onset CRC and gynecological cancers, fulfilling the Amsterdam II criteria for Lynch syndrome [20, 21]. This variant has been classified as pathogenic in ClinVar and other curated databases. As shown in Table 2, MSI testing indicated microsatellite instability, and immunohistochemistry (IHC) analysis consistently revealed MMR deficiency in the patient from Family 1 (II‐6), further supporting the diagnosis of Lynch syndrome in this family.

Previously conducted research, such as that by Chang et al. [22], has underscored the significant role of *MLH1* variants in CRC risk, particularly in Asia. This study reports *ERBB2* (p.W9fs), R385C, and T117M variants in *MLH1* among Taiwanese CRC patients. The discovery of the new c.1043T>A (p.L348X) variant in *MLH1* further validates the critical role of MMR genes in CRC, advancing knowledge in the field of pathogenic variants.

In addition, in a study carried out by Singh AK et al. [23], WES of 48 familial CRC patients identified one suspicious variant of uncertain significance (VUS) in the *MLH1* gene (c.514G>A, p.Glu172Lys) in two affected members of a family. Despite its VUS classification, multiple lines of evidence suggested its potential pathogenicity. Also, in a large‐scale study utilizing GWAS and whole‐exome sequencing data from 3362 CRC cases and over 133,000 controls, *MLH1* was identified as a significant CRC susceptibility gene. Pathogenic variants classified as loss‐of‐function (pLOF) in *MLH1* were strongly associated with increased CRC risk (*p* = 1.35 × 10^−7^) [24]. In addition, in our previous study, we identified two (likely) pathogenic heterozygous variants, c.1077‐2A>G and c.199G>A, in the *MSH2* and *MLH1* genes, respectively, in two unrelated Iranian families [25].

In another study, Wang et al. [26] reported *MLH1*‐related genetic variants in CRC, focusing on tumors with *MLH1* promoter hypermethylation (MLH1me+). They found that MLH1me + CRCs frequently harbored RNF43 variants (73.1%) and BRAF V600E (38.8%), but rarely KRAS, APC, or CTNNB1 alterations. Notably, RNF43 variants were independently associated with MLH1me + status, suggesting a unique molecular subtype. Their work highlights the importance of stratifying dMMR CRCs by *MLH1* alterations for targeted therapies.

Furthermore, researchers, including Yunos et al. [27], have identified a spectrum of *ERBB2* variants, including nonsynonymous alterations (R648Q and V84M), in CRC, specifically in proximal colon tumors. Another study conducted by Afolabi HA et al. [17] reported the c.2632C>T (p.His878Tyr) variant in *ERBB2* at position 37,881,440 in GRCh37 on chromosome 17, a missense variant that resulted in an amino acid substitution from Histidine to Tyrosine at protein position 878 and was identified as a deleterious/damaging clinical implication. However, the *ERBB2* gene was classified as “variant unspecified” in the databases. Other studies have also pointed to possible connections between *ERBB2* variants and CRC, supporting the link between *ERBB2* amplifications and variants and CRC aggressive forms, as well as poor prognosis. In the meantime, the 2017 GENIE consortium data reported *ERBB2* alterations in a small but significant percentage of CRC cases, underscoring its potential as a therapeutic target.

In the meantime, the 2017 GENIE consortium data reported *ERBB2* alterations in a small but considerable percentage of CRC cases, emphasizing its possible contributions as a therapeutic target [28]. Nevertheless, previous research has not documented the specific variants *ERBB2* c.2268G>T (p.R756S) and *MLH1* c.320T>A (p.L107X) in CRC populations, although this study offers promising insights into CRC's genetic landscape among Iranians.

The *MLH1* gene comprises 19 exons and encodes a 756‐amino‐acid protein, Human MutL Homolog 1 (Mlh1), which participates in the mismatch repair process [29]. MMR represents a highly conserved mechanism primarily responsible for correcting base–base mismatches and insertion–deletion loops during DNA replication, thereby enhancing replication fidelity by up to 3 orders of magnitude. MMR‐related heterozygous germline inactivation results in Lynch syndrome, which is mainly a hereditary cancer predisposition predominantly affecting the endometrium and colorectum but also influencing other organs. Lynch syndrome accounts for 2%–5% of all CRCs, although nearly 15% of sporadic CRC cases also exhibit somatic MMR inactivation, mainly due to *MLH1* promoter hypermethylation [30]. Over 3000 pathogenic alterations in MMR genes have been documented in the ClinVar database, with the majority reported for *MLH1* and *MSH2* [29].

Approximately 2% of all CRCs exhibit *ERBB2* (also known as HER2) amplification. ERBB2 belongs to the EGFR family, consisting of ERBB1 (EGFR), ERBB3, and ERBB4. Similar to other EGFR family members, this tyrosine kinase receptor comprises extracellular, transmembrane, and cytoplasmic domains [31]. *HER2*, a proto‐oncogene on chromosome 17q21, lacks ligand‐binding activities but is capable of forming homo‐ or hetero‐dimers at the cell membrane with other members of the EGFR family, such as HER1/EGFR, HER3, HER4, initiating tyrosine kinase domain transphosphorylation, key signaling pathway activation, including phosphoinositide 3 kinase (PI3K)/Akt/mammalian target of rapamycin (mTOR), transducing and activating factor of transcription (JAK/STAT) pathway, RAS–RAF‐mitogen activated protein kinase/extracellular signal‐regulated kinase (MAPK/ERK) pathway, and phospholipase C (PLC)/protein kinase C (PKC) pathway. Cell proliferation, survival, and migration are regulated through these pathways [32]. *HER2* receptor overexpression influences various cellular processes, including invasion, migration, differentiation, angiogenesis, and chemoresistance, making it a highly predictive biomarker for tracking the progression of invasive clinical diseases [33]. Different ERBB2 amplification/HER2 overexpression rates have been reported at 3%–47.4% in CRC, underscoring the limited understanding of HER2's clinical relevance in CRC [34, 35, 36]. Meanwhile, relevant studies support an approximately 5% frequency of *ERBB2* alterations in CRC cases, raising the possibility that *ERBB2* can be a valuable target for anti‐ERBB2 therapies and prognostic investigations [37].

Structural modeling of the MLH1 and ERBB2 proteins using AlphaFold3 has provided crucial insights into the pathogenic mechanisms associated with identified variants. The MLH1 L348X variant introduces a premature stop codon, resulting in truncation of the protein and loss of its C‐terminal domain, which is essential for interaction with PMS2 and the formation of the MutLα complex. This truncation disrupts the DNA mismatch repair system, as evidenced by a significant decrease in binding affinity (from −40.1 to −24.1 kcal/mol). This reduction indicates that MLH1 dysfunction contributes to microsatellite instability and tumorigenesis [10]. These findings are consistent with previous reports that have highlighted the importance of the C‐terminal domain in maintaining the integrity of MutLα and its role in mismatch repair [13].

The *ERBB2* R756S missense variant identified in Family 2 is a compelling candidate for pathogenicity due to its location within the critical tyrosine kinase domain, where it is predicted to directly impact the ATP‐binding site. The substitution of a bulky, positively charged arginine with a small, uncharged serine at this evolutionarily conserved position is likely to disrupt ATP coordination and alter kinase conformation, potentially leading to constitutive activation—a well‐established gain‐of‐function mechanism for ERBB2 in oncogenesis. The variant's significance is further underscored by its proximity to the L755 residue, a known hotspot for tyrosine kinase inhibitor resistance (e.g., lapatinib) [38]. Although the p.Arg756Ser variant itself is not frequently reported, its recurrence as a somatic mutation in the COSMIC database (COSM1025801) and its position within this key functional domain strongly suggest a role in altered signaling and oncogenic transformation [16]. Despite its current classification as a VUS in ClinVar, our evidence supports a re‐classification to Likely Pathogenic according to ACMG/AMP guidelines. This assessment is based on the following criteria: its absence in population databases (PM2), location in a well‐established mutational hotspot and critical functional domain (PM1), and concordant deleterious predictions from multiple in silico tools (PP3). While the variant shows incomplete penetrance, as evidenced by its presence in the unaffected father (II‐4, age 68) and brother (III‐2, age 42) of the proband, the weight of the combined molecular evidence within the ACMG framework supports pathogenicity. This classification is crucial for guiding clinical management and surveillance of carriers and for highlighting the importance of periodic re‐evaluation of variants as new evidence emerges.

A study by Le et al. in an Asian cohort reported that more than 94% of early‐onset colorectal cancer (EOCRC) cases were associated with somatic mutations in common cancer‐related genes, including *TP53*, *APC*, and *KRAS*. These genes showed the highest mutation frequency, indicating their central role in CRC development in this population. In comparison, our study found novel mutations in *MLH1* (c.1043T>A) and *ERBB2* (c.2268G>T), which seemed to be linked to familial CRC at an early age. The findings highlight the need to explore rare genetic variations in the Iranian population and in other Middle Eastern cohorts [39].

Malibary et al. noted that recent developments in CRC screening, particularly in the Euro‐Asian region, reflect the implementation of blood‐based biomarkers and molecular techniques. These methods, combined with traditional colonoscopy, are expected to increase the rates of early cancer diagnosis and achieve better clinical results. These findings align with the implications of our own research for genetic screening, suggesting that incorporating genomic data into CRC screening may be of significant value to high‐risk populations, especially those with a family history of early disease [40].

The current paper was primarily limited by its fairly small sample size, focusing on only two Iranian families with CRC records. Although the study offers valuable insights into potential genetic variants among these patients, the findings can be further validated in a larger cohort, enabling assessments of the identified alterations in terms of their broader applicability. Another limitation of this study is the lack of functional validation for the identified *ERBB2* p.R756S variant. While in silico models strongly support a deleterious effect, future work should include immunohistochemical analysis of ERBB2 expression and phosphorylation status, as well as assessment of downstream MAPK and PI3K‐AKT signaling, to experimentally confirm the variant's impact on receptor function and its role in oncogenesis. In addition, the study relied mainly on WES, which may lead to the loss of certain genetic variations, including those in the genome's non‐coding regions. However, it has been recognized as a comprehensive technique. It is also worth noting that the study did not address other potential environmental factors contributing to the CRC development, which might consequently affect the overall interpretation of the research results. Ultimately, although functionally validating the discovered alterations and their direct impacts on cancer development would provide a thorough understanding of their clinical relevance, the current research did not focus on this issue, leaving future researchers to conduct broader investigations and address this limitation.

## 7 Conclusion

The genomic technique known as WES has revealed complex and heterogeneous genetic applications for addressing CRC issues. The identification of gene variants such as *MLH1* and *ERBB2* has provided valuable insights into the biological profile of CRC, laying the groundwork for precision or personalized medicine approaches. The findings from this research underscore the critical importance of integrating genomic data into clinical practice to improve diagnostic, prognostic, and therapeutic strategies for patients with CRC. As WES becomes increasingly accessible, its essential role in discovering new biomarkers and therapeutic targets will continue to grow, indicating promising advancements in more precise and tailored cancer care.

## Ethics Statement

All patients gave written informed consent in accordance with the Declaration of Helsinki. The study protocol was approved by the Ethics Committee of the Research Center for Gastroenterology and Liver Diseases (RCGLD) (Tehran, Iran). The accession numbers for the variants c.2268G>T and c.1043T>A reported in this paper are [ClinVar]: [SCV006082354] and [SCV006082353], respectively.

## Conflicts of Interest

The authors declare no conflicts of interest.

## Data Availability

The datasets generated and/or analyzed during the current study are available in the ClinVar repository at the following links: [https://www.ncbi.nlm.nih.gov/clinvar/variation/1399145/?oq=SCV006082354&m=NM_004 448.4(ERBB2):c.2268G%3ET%20(p.Arg756Ser)] and [https://www.ncbi.nlm.nih.gov/clinvar/variation/2674448/?oq=SCV006082353&m=NM_000 249.4(MLH1):c.1043T%3EA%20(p.Leu348Ter)]. The accession numbers of the variants in ClinVar are as follows: NM_004448.4(ERBB2): c.2268G>T (p.Arg756Ser): VCV001399145.8 NM_000249.4(MLH1): c.1043T>A (p.Leu348Ter): VCV002674448.4.
